# The Role of 3D-pCASL MRI in the Differential Diagnosis of Glioblastoma and Brain Metastases

**DOI:** 10.3389/fonc.2022.874924

**Published:** 2022-04-26

**Authors:** Kristina Solozhentseva, Artem Batalov, Natalia Zakharova, Sergey Goryaynov, Eduard Pogosbekyan, Igor Pronin

**Affiliations:** N.N. Burdenko National Medical Research Center of Neurosurgery, Ministry of Health of the Russian Federation, Moscow, Russia

**Keywords:** glioblastoma, brain metastases, blood flow, peritumoral zone, intratumoral blood flow

## Abstract

**Purpose:**

The first aim of this study was to compare the intratumoral and peritumoral blood flow parameters in glioblastomas and brain metastases measured by pseudocontinuous arterial spin labeling MRI (3D pCASL). The second aim of this study was to determine whether pCASL could aid in identifying the source of brain metastases.

**Materials and Methods:**

This study included 173 patients aged 12 to 83 years (median age—61 years), who were observed at the National Medical Research Center for Neurosurgery. All patients underwent preoperative MRI with pCASL perfusion. Thereafter patients were operated on and received histological diagnosis. No patients received preoperative chemo or radiotherapy.

**Results:**

The values of maximum and normalized intratumoral blood flow were significantly higher in the group with gliblastoma than in the group with brain metastases: 168.98 + −91.96 versus 152.1 + −173.32 and 7.6 + −8.4 versus 9.3 + −5.33 respectively (p <0.01). However, ROC analysis showed low AUC specificity and sensitivity (0.64, 70%, 60% for mTBF and 0.66, 77%, 62% for nTBF). Peritumoral blood flow parameters were also higher in the glioblastoma group (29.61 + −22.89 versus 16.58 + −6.46 for mTBF and 1.63 + −1.14 versus 0.88 + −0.38 for nTBF, respectively; p <0.01). ROC analysis showed the following measurements of AUC, specificity, and sensitivity (0.75, 68%, 73% for mTBF and 0.77, 58%, 91% for nTBF). Regarding pCASL and various histological subsets of brain metastases, the study found statistically significant differences between the lung and melanoma metastases and the lung and kidney metastases. ROC analysis gave the following values for lung and melanoma metastases: AUC—0.76, specificity—75%, and sensitivity—73% for mTBF; 0.83, 67%, and 93% respectively, for nTBF. For lung and kidney metastases: AUC—0.74, specificity—70%, and sensitivity—93% for mTBF; 0.75, 70%, and 93% respectively, for nTBF.

**Conclusions:**

pCASL could aid in differential diagnosis between glioblastoma and brain metastases. Measurement of peritumoral blood flow demonstrates higher specificity and sensitivity than with intratumoral blood flow. Moreover, pCASL provides the ability to distinguish lung metastases from kidney and melanoma metastases.

## Introduction

**Figure 1:**
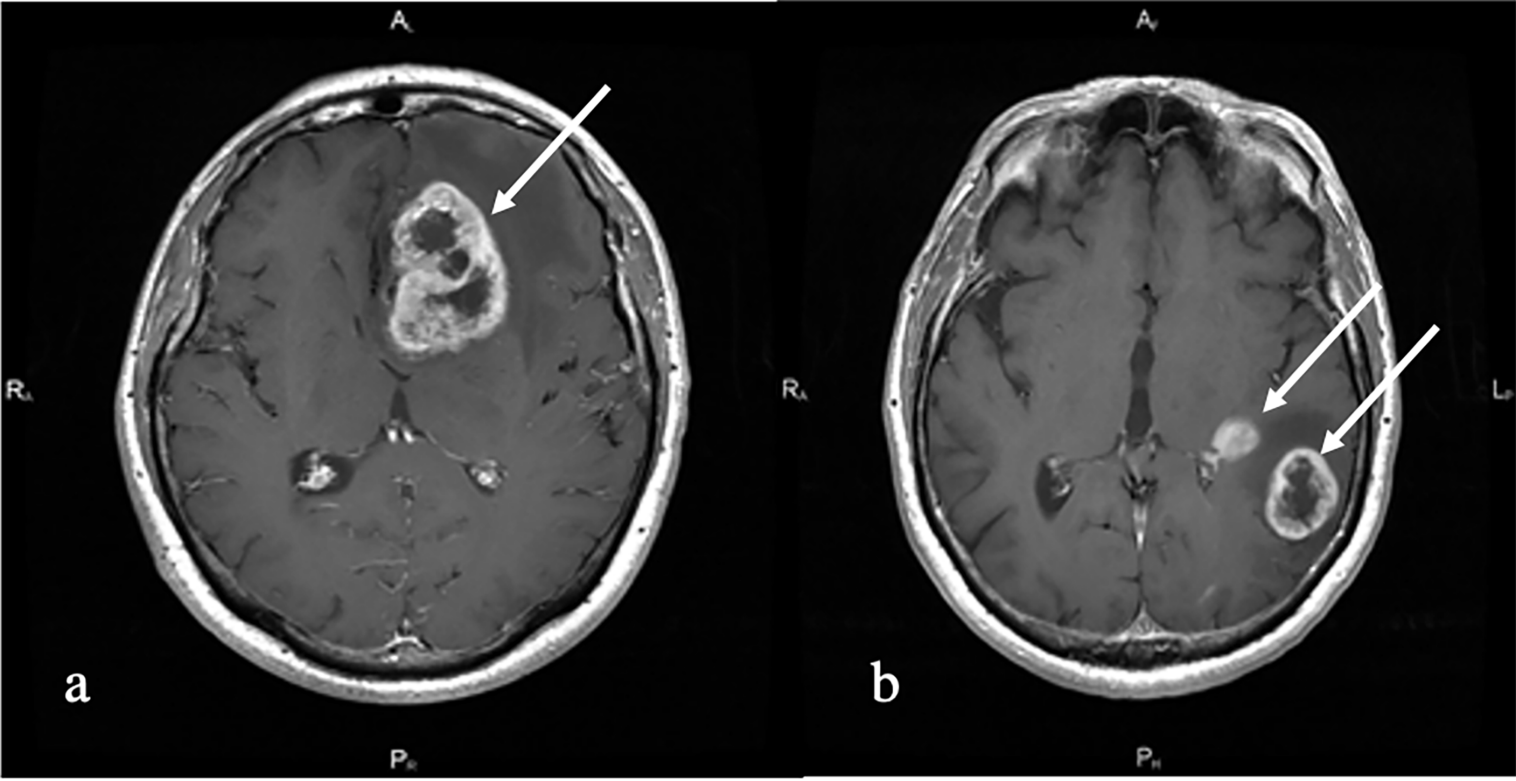
T1 with contrast enhancement. **(A)** Melanoma metastases (white arrow). Left frontal lobe solitary irregularly contrast-enhancing tumor with central non-enhancing areas and perifocal edema is shown. **(B)** Multiple primary glioblastoma (white arrows). Left temporal multiple irregularly contrast-enhancing lesions with perifocal edema and non-enhancing central part are shown.

The differential diagnosis of metastatic brain lesions and malignant gliomas is an extremely important task, since further approaches to the diagnosis and treatment of these patients differ significantly. Current methods for differentiating glioblastoma from brain metastases are primarily based on the multiplicity of brain metastases, however 50% of metastases are single lesions (1) (**Figure 1**). Even worse, there are multicentric glioblastomas (2). Secondly, location is important. Metastases rarely involve the corpus callosum and tend to be located gray and white matter junctions, whereas glioblastomas tend to be centered in the white matter (3). Morphologically, the presence of infiltration characterizes glioblastoma—small growths of tumor tissue along the white matter (3). In addition to that, there is an essential feature—glioblastoma can have non-enhancing tumor tissue, which is not typical for metastatic brain damage (4).

To conclude, making a differential diagnosis between glioblastoma and brain metastases using only standard MRI sequences alone could be a challenging task (5).

In terms of advanced MRI technology, MRI-perfusion and MRI-spectroscopy play a significant role in differentiating these two entities. This study explores the role of ASL-perfusion.

Previously, studies of intratumoral and peritumoral blood flow using contrast MR perfusion (DSC) were conducted, which showed that the values of blood flow in the peritumoral zone were significantly higher for glioblastomas (6). However these studies did not reveal statistically significant differences in intratumoral blood flow (7–9). pCASL MRI-perfusion is a quantitative method for non-contrast assessment of blood flow (10). This method is widely used in the examination of patients with brain tumors, but its role in the differential diagnosis of metastatic lesions and glioblastomas has not been studied enough (11, 12).

Another diagnostic task to determine the histological subtype of brain metastases. Distinguishing different types of brain metastases by blood flow may be clinically important, especially in cases when PET-CT is not available. The assumption of the source of metastases by MRI can affect the order in which diagnostic tests are applied to a patient.

Attempts have been made to differentiate metastases using MR spectroscopy, MR diffusion and CT perfusion (13, 14). The sensitivity and specificity of these methods were found to be low. The use of MR spectroscopy made it possible to distinguish melanoma metastases from all other histological subgroups (15). In the SWI study, it was shown that with quantitative analysis it is possible to differentiate metastases of melanoma from metastases of breast and lung cancer. In the study of diffusion-weighted MRI, no statistically significant differences in diffusion coefficients were found for different histological subtypes of metastases (16).

The study evaluated the role of pCASL in the differential diagnosis of metastases and glioblastomas and studied the blood flow in various histological subtypes of metastases.

## Materials and Methods

This study included 173 patients aged 12 to 83 years (median age—61 years) who were observed at the National Medical Research Center for Neurosurgery from 2012 to 2020. Fifty-two percent were women, and 48% were men. The study was retrospective. The inclusion criteria for the study were: 1. All patients must have a diagnosis of either glioblastoma or brain metatastatic disease; 2. All patients must have an MRI with T2-FLAR, T1 after contrast enhancement, and pCASL-perfusion performed at our hospital before surgery. 3. All patients must have had histological verification, which was done by our pathology department.

Of the 173 patients, 55 were diagnosed with brain metastases and 118 with glioblastomas. All patients underwent further tumor resection or stereotactic biopsy with subsequent histological verification of the process. In the group with Brain metastases the following subgroups/subtypes depending on the primary tumor source were established: melanoma (n = 12), kidney cancer (n = 10), breast cancer (n = 12), lung cancer (n = 15), intestinal cancer (n = 4), other tumors (n = 2). The subgroup “other tumors” included patients with diagnoses of metastases of myeloid sarcoma and metastases of cancer of unknown primary origin.

MRI was performed on a 3.0 T General Electric Signa HD MR tomography (GE Healthcare) with an 8-channel head coil. The MRI protocol for all patients consisted of T2, T2-FLAIR, DWI, 3D pCASL, and T1 before and after contrast enhancement.

ASL-scanning was carried out with the following parameters: 3D FSE, 8-lead spiral scanning with the capture of the entire volume of the brain and subsequent reforming with a section thickness of 4 mm; FOV = 240 × 240 mm; matrix 128 × 128, ZIP 512; TR—4717 ms; TE—9.8 ms; NEX = 3; PLD—1525 ms; pixel bandwidth—976.6 Hz/pixel. The duration of scanning was 4 min 30 s.

The ReadyView software package (GE Healthcare) was used to post-process the obtained data

The absolute maximum blood flow in the tumors was measured in all patients, and normalized values were calculated. To do this, an experienced neuroradiologist (>5 years of experience) placed the 4–5 ROIs (Region of Interest) of a small area (20 ± 10 mm^2^) on a color TBF map in every slice within the tumoral stroma. Then the ROI with the highest blood flow was chosen.

Normalization of blood flow values was carried out by dividing the TBF value in the tumor and peritumoral zone by the TBF value in the unchanged white matter of the contralateral side.

In all patients, the values of blood flow in the peritumoral zone were calculated. The ROI area was 20 ± 10 mm^2^. All patients received three ROIs in the peritumoral zone at the same level where the intratumoral ROI was placed: ROI1 was drawn in the area of increased MR signal in T2-FLAIR, where there was no accumulation of contrast agent, close to the contrasted part of the tumor (no further than 5 mm). ROI3 was drawn in the area of increased MR signal in T2-FLAIR, at the greatest distance from the contrasted part of the tumor, close to the unchanged white matter, and ROI2 was indicated at an equal distance between ROI1 and ROI3.

In the group of metastases and glioblastomas, a comparison was made of tumor blood flow parameters, ROI1 values, and ROI1–ROI3 gradient values, followed by ROC analysis. The gradient was calculated by subtracting the TBF values at ROI3 from the TBF values at ROI1.

Also, we performed a comparison between the maximum blood flow and normalized values in the histological subtypes of metastases.

The Neuro registration program was used to align blood flow maps with anatomical images (T2-FLAIR, T1 with contrast enhancement).

Statistical processing was carried out in the R-project program; the pROC library was used for ROC analysis. The Spirimen method was used to calculate the correlation coefficients. The Mann–Whitney U test was used to evaluate group comparisons.

## Results

**Figure 2:**
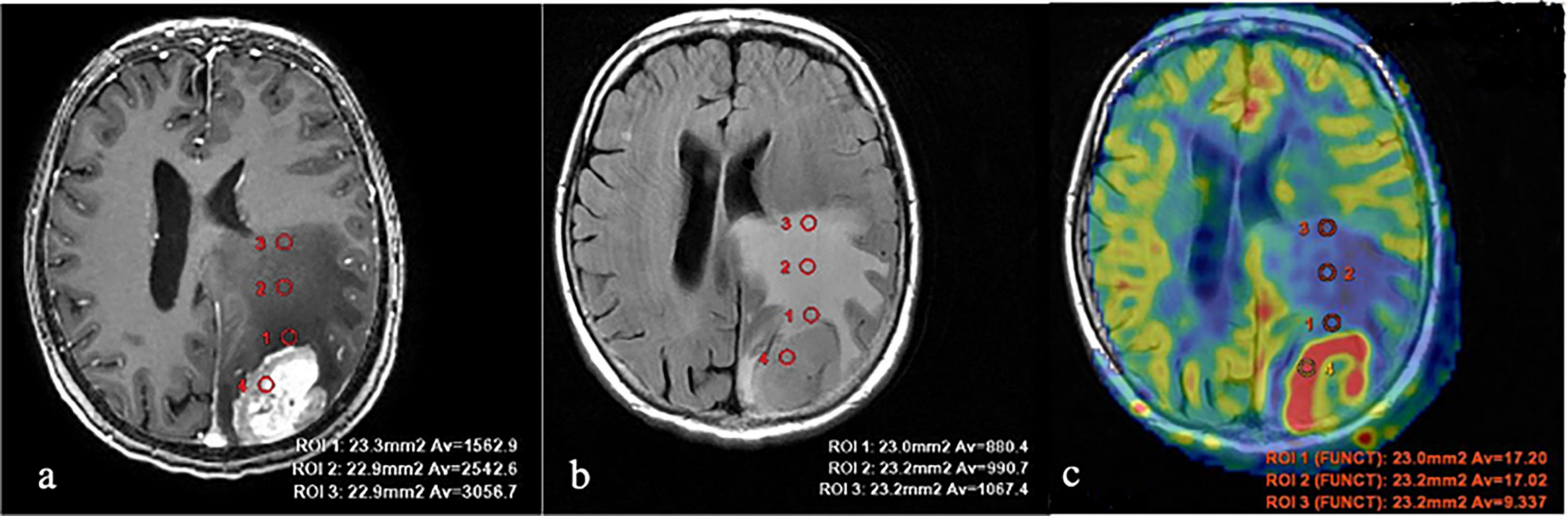
Breast cancer metastases. **(A)** T1 with contrast enhancement; **(B)** T2-FLAIR; and **(C)** TBF blood flow maps aligned with T2-FLAIR. Measurements of hemodynamic parameters in the tumor and peritumoral zone are shown.

**Figure 3:**
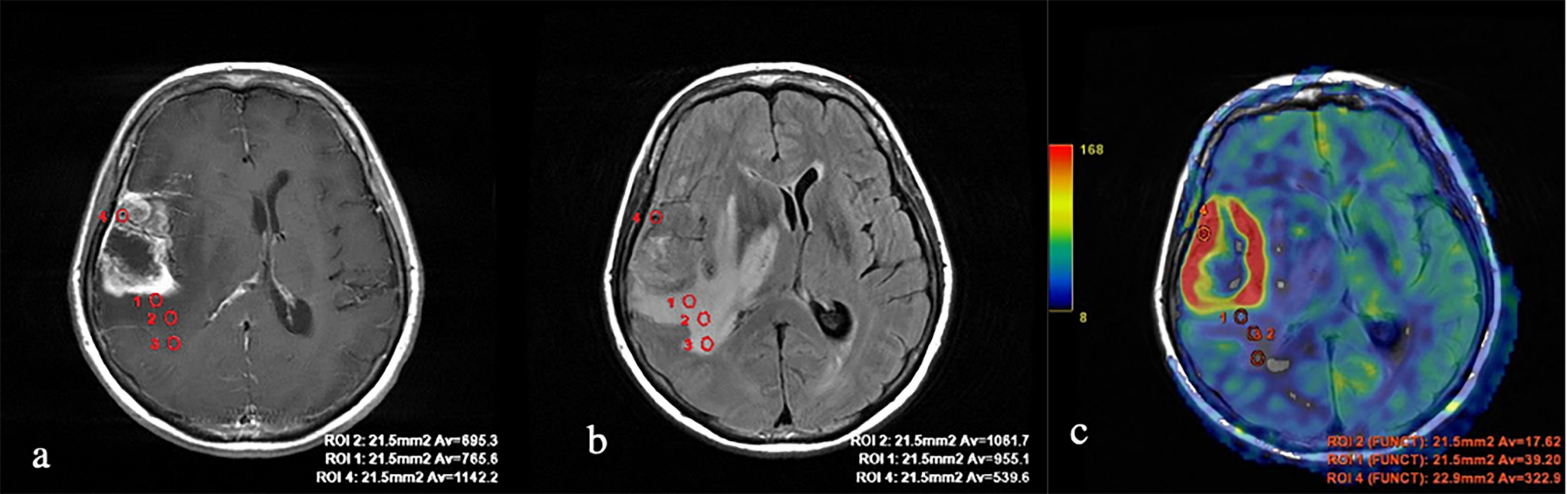
Glioblastoma. **(A)** T1 with contrast enhancement; **(B)** T2-FLAIR; and **(C)** TBF blood flow maps aligned with T2-FLAIR. Measurements of hemodynamic parameters of tumor blood flow in the peritumoral zone are shown.

**Figure 4:**
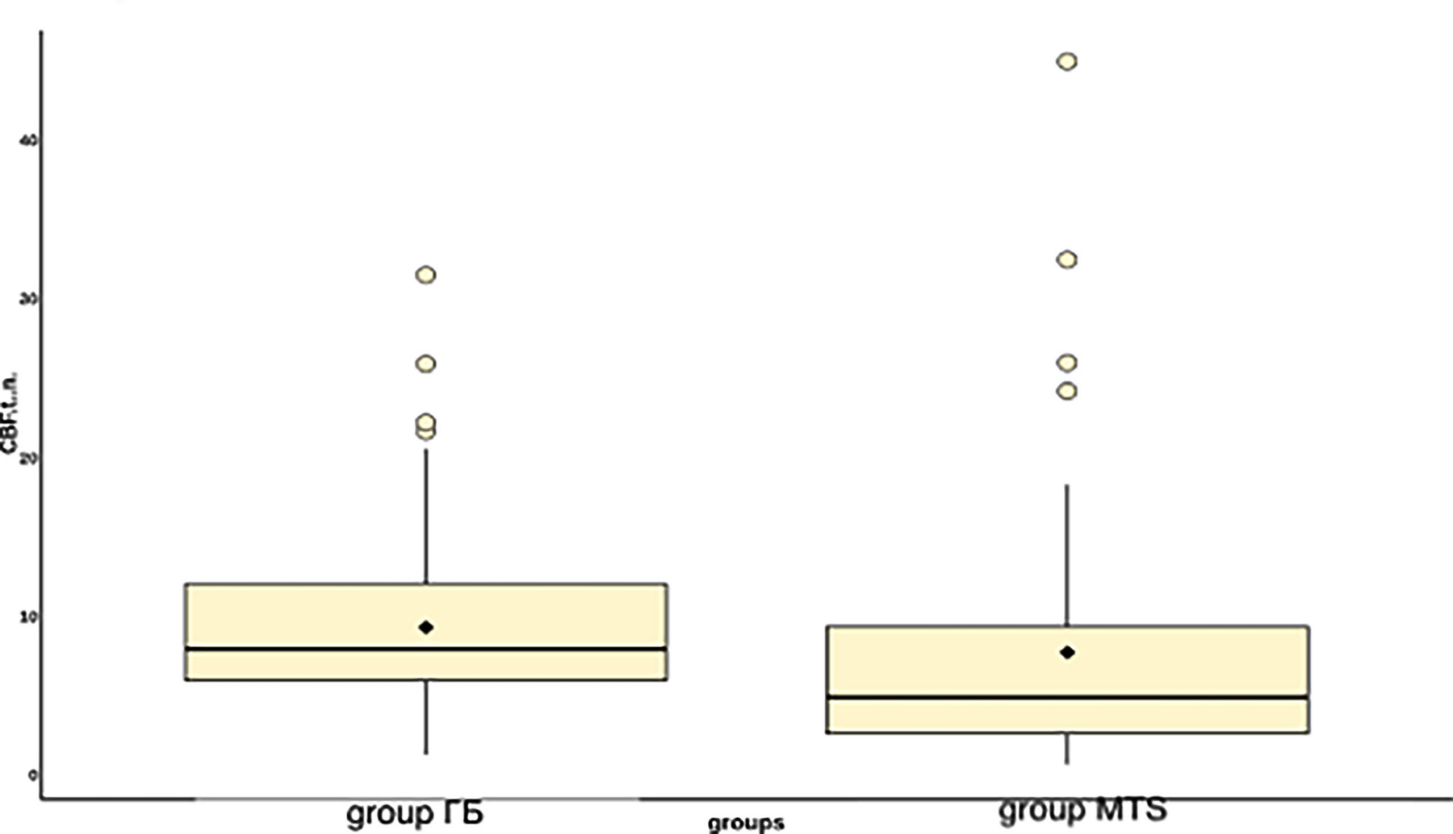
Boxplot displaying blood flow values in groups of metastases and glioblastomas.

First, we obtained the average absolute and normalized values of tumor blood flow in the groups of metastases and glioblastomas. TBF measurements in metastatic brain disease (**Figure 2**) were statistically lower than in glioblastomas (**Figure 3**) (p-value *<*0.05). The data are presented in **Table 1** and **Figure 4**.

**Table 1:** Average values of blood flow in metastatic and glioblastoma subgroups.

Group	Maximum absolute TBF t	Standard deviation TBF t	Maximum normalized TBF t (norm)	Standard deviation TBF t (norm)
Metastases	152.1	173.32	7.6	8.4
Glioblastoma	168.98	91.96	9.3	5.33

TBF, cerebral blood flow.

**Figure 5:**
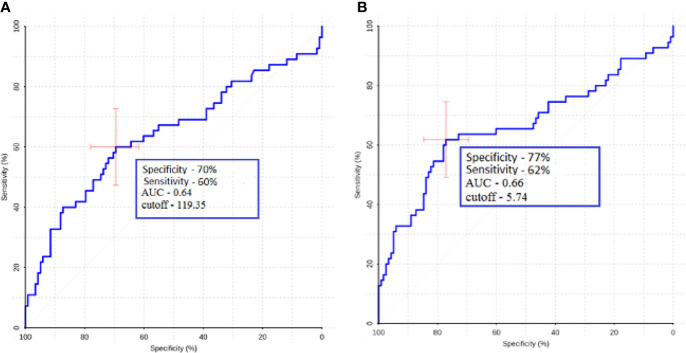
ROC analysis data obtained by comparing the maximum **(A)** and normalized values **(B)** of intratumoral blood flow in the groups of metastases and glioblastomas. The values of the optimal threshold are presented, in brackets—specificity and sensitivity.

ROC analysis was performed to determine the sensitivity and specificity of ASL perfusion in the differential diagnosis of glioblastomas and secondary brain damage. The ROC analysis data are presented in **Figure 5**.

**Figure 6:**
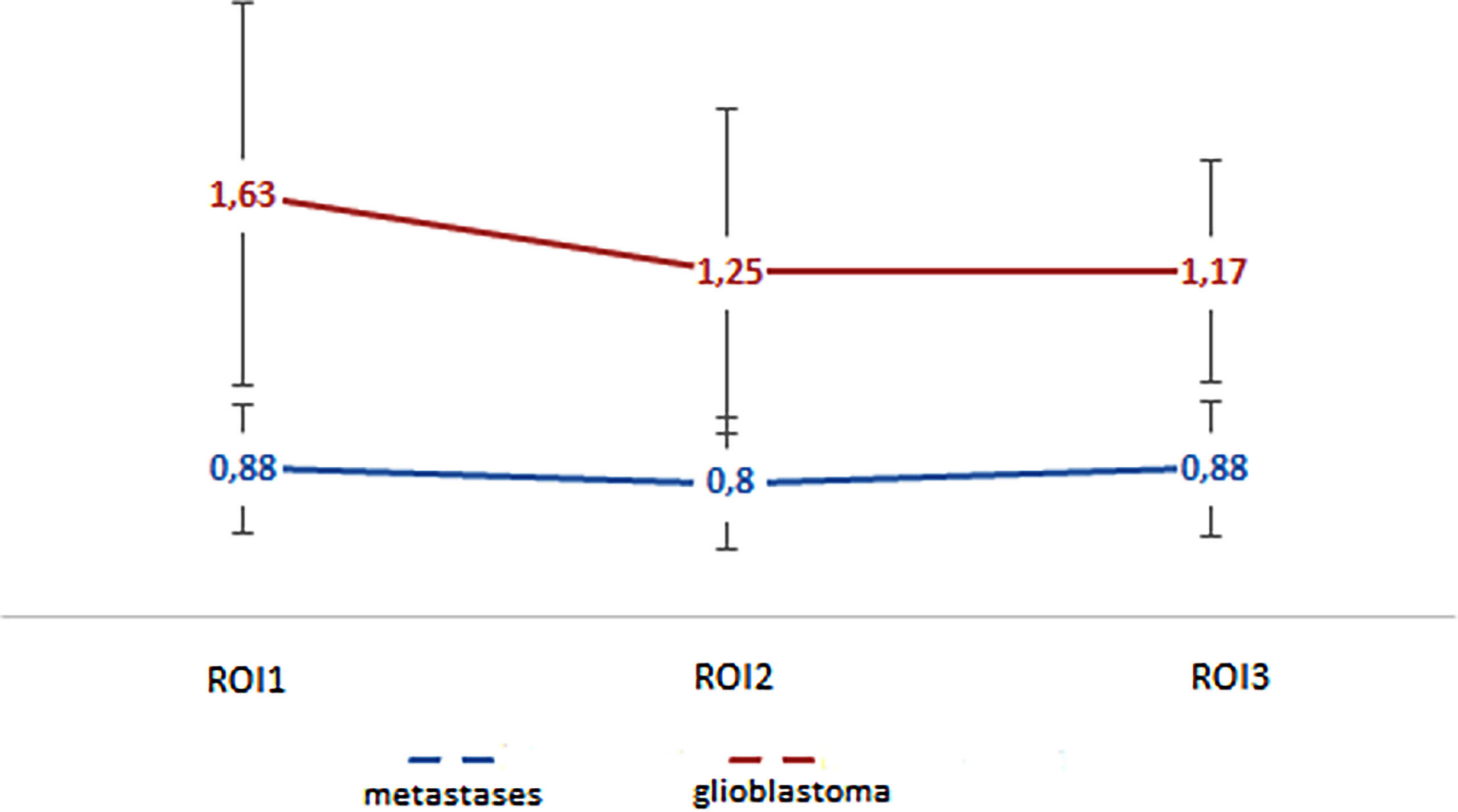
Changes in blood flow indicators in areas of interest: edema (metastases) and edema with infiltrative component (glioblastoma).

In our work, the mean absolute and normalized values of blood flow in the peritumoral zone of groups of glioblastomas and metastases were calculated (**Table 2**). When comparing absolute and normalized parameters (ROI1), peritumoral blood flow in the glioblastoma group was significantly higher (p-value <0.0001). Simultaneously, there were no significant differences in the values of ROI2 and ROI3. However, it was noticed that the blood flow in the peritumoral zone of glioblastomas toward the unchanged white matter decreases and does not change with metastatic brain damage. These results are illustrated in **Figure 6**.

**Table 2:** Average values of absolute and normalized indices of peritumoral blood flow in the groups of metastases and glioblastomas.

Group	Maximum absolute TBF ROI1	Maximum normalized TBF ROI1	Maximum absolute TBF ROI2	Maximum normalized TBF ROI2	Maximum normalized TBF ROI3	Maximum normalized TBF ROI3
Metastases	16.58 ± 6.46	0.88 ± 0.38	15.53 ± 6.67	0.8 ± 0.39	17.15 ± 4.13	0.88 ± 0.4
Glioblastoma	29.61 ± 22.89	1.63 ± 1.14	23.45 ± 21.24	1.25 ± 0.96	21.98 ± 11.67	1.17 ± 0.65

TBF, tumor blood flow; ROI, region of interest.

**Figure 7:**
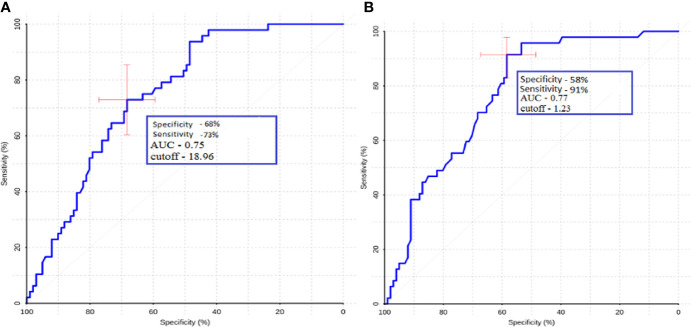
ROC-analysis data obtained by comparing the maximum absolute **(A)** and normalized **(B)** TBF values at the ROI1 point in the groups of metastases and glioblastomas. The values of the optimal threshold are presented, in brackets—specificity and sensitivity.

For ROI1 values, ROC analysis was carried out, which showed rather high sensitivity and specificity values. The area under the curve was 0.75 and 0.77 for absolute and normalized values, respectively, which indicates good diagnostic capabilities of TBF measurement in the peritumoral zone (**Figure 7**).

**Figure 8:**
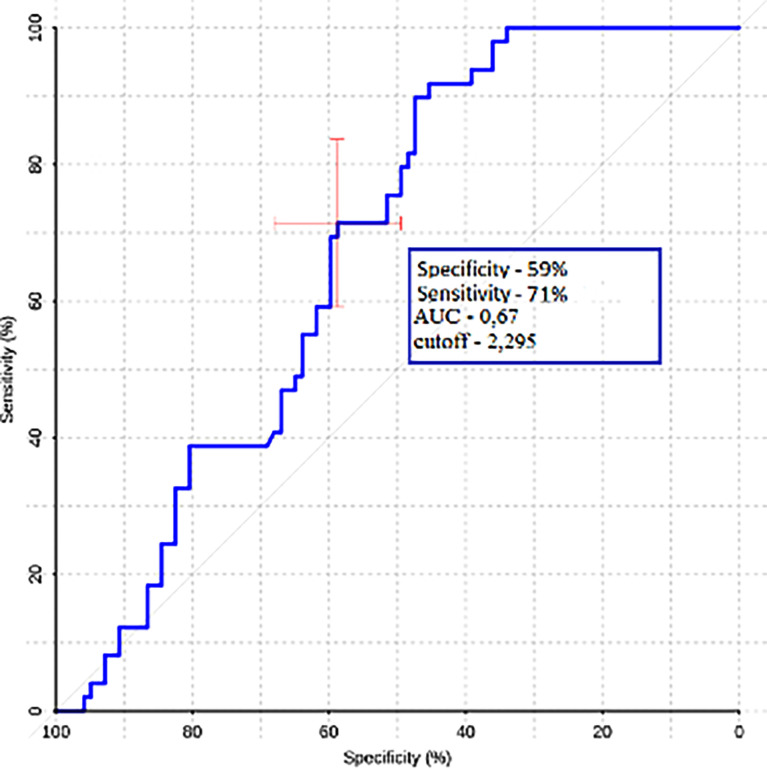
ROC-analysis data obtained by comparing the maximum absolute values of the ROI1–ROI3 gradient in the groups of metastases and glioblastomas.

Gradient values (ROI1–ROI3) were also calculated for the group of metastases and glioblastomas (**Table 3**). The mean ROI–ROI3 gradient for the group of metastases turned out to be negative, which indicates lower values of blood flow in the white matter near the contrasted part of the tumor. It was proven that there was a statistically significant difference between glioblastoma and metastases (p-value was 0.0008). A comparative analysis of ROI1–RO3 gradients was carried out. The area under the curve was 0.67 (**Figure 8**).

**Table 3:** Mean values of peritumoral blood flow gradient (ROI1–ROI3) for metastases and glioblastomas.

Group	Maximum absolute TBF ROI1–ROI3	ROI1–ROI3 standard deviation
Metastases	−0.82	6.03
Glioblastoma	7.63	11.22

**Figure 9:**
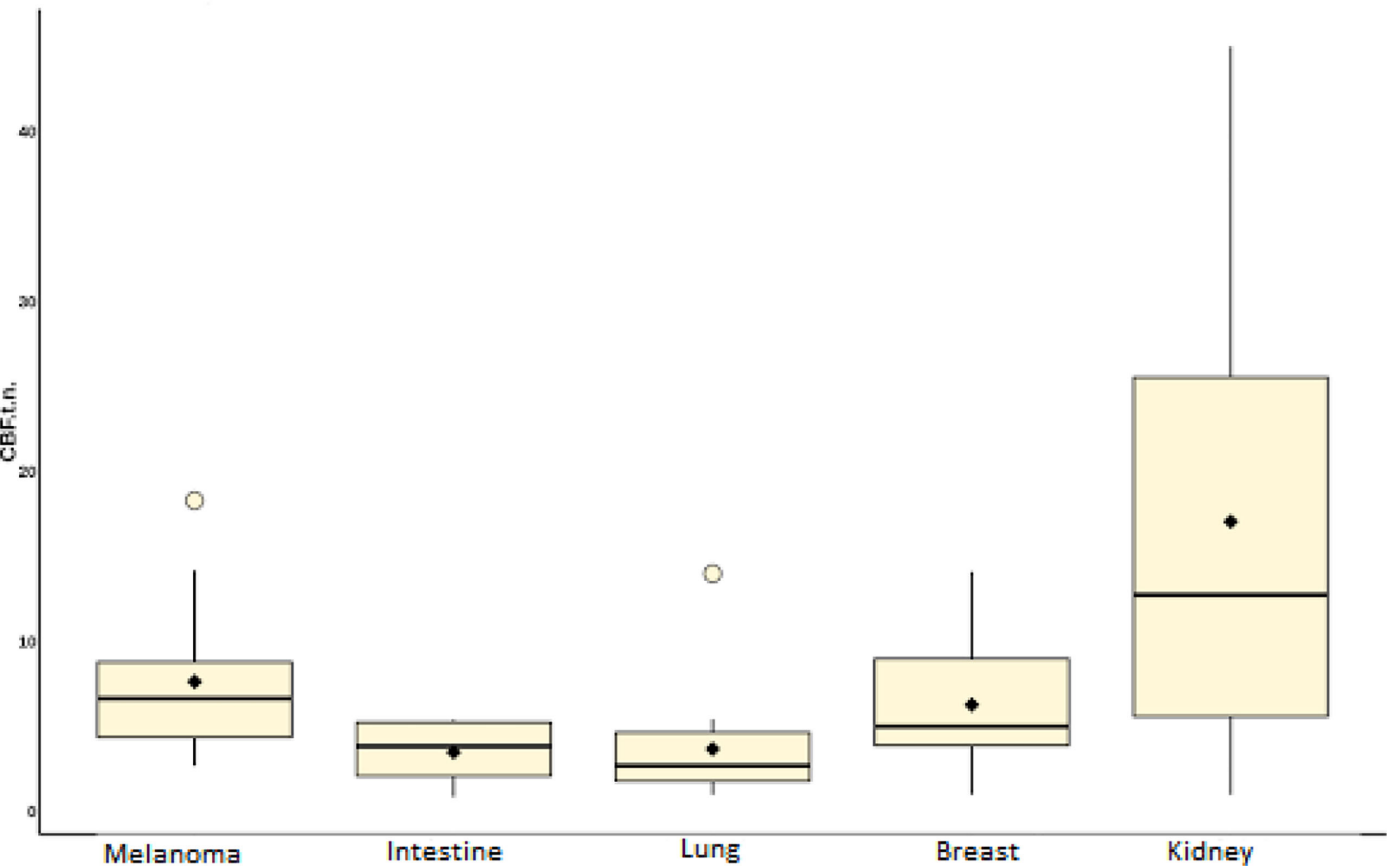
Box plot showing the value of intratumoral blood flow in the group of metastatic brain lesions.

In the second part of our work, a comparative analysis of intratumoral blood flow in histological subgroups of metastatic brain lesions was carried out. Average values of TBF for metastases of the kidney, melanoma, breast, lung, and intestine are presented in **Table 4** and **Figure 9**.

**Table 4:** Mean values of tumor blood flow in different subtypes of metastatic lesions.

Group	TBF (melanoma)	TBF (lung)	TBF (kidney)	TBF (intestines)	TBF (breast)
maxTBF	148.43	82.19	331.46	66.62	138.42
Standard deviation	88.32	65.05	326.03	7.35	81.91

MaxTBF, maximal TBF.

Intratumoral TBF was shown to be statistically lower in the subgroup of lung cancer metastases compared to the metastatic kidney and melanoma groups (p <0.05). In the subgroups of the lung and breast, statistically significant differences were also obtained. However, when analyzing the normalized values, the p-value was 0.09.

The lowest mean blood flow values were obtained in the subgroup of intestinal cancer metastases. However, when performing a comparative analysis with all other histological types, no significant differences were obtained (p-value >0.05), which is probably due to a small sample of patients with metastases of intestinal cancer (n = 4).

ROC analysis was performed for groups that showed statistically significant differences.

**Figure 10:**
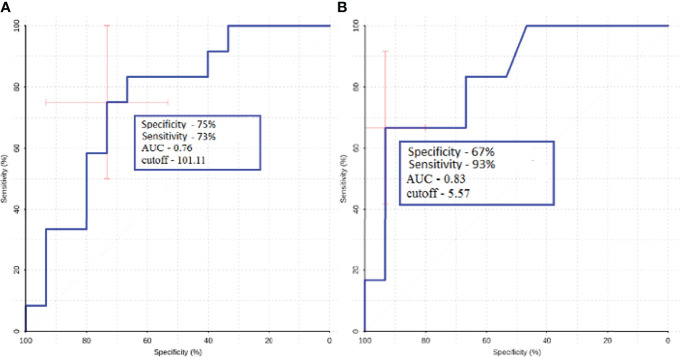
ROC-analysis data obtained when comparing the maximum absolute **(A)** and normalized **(B)** values of intratumoral blood flow in the groups of metastatic lesions in lung cancer and melanoma.

ROC-analysis showed high sensitivity and sensibility in melanoma and lung cancer metastases (**Figure 10**).

**Figure 11:**
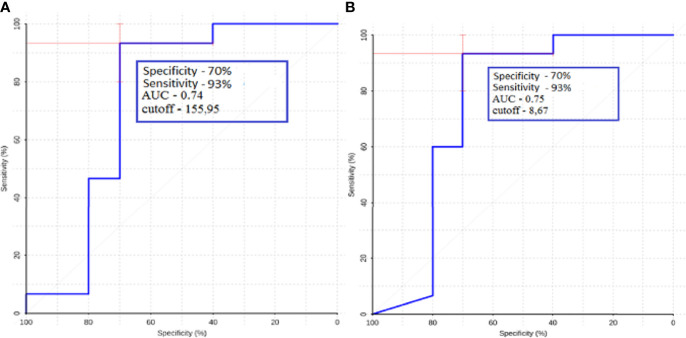
ROC-analysis data obtained when comparing the maximum absolute **(A)** and normalized **(B)** values of intratumoral blood flow in the groups of metastatic lesions in lung cancer and kidney cancer.

ROC analysis was performed for metastatic subgroups of lung cancer and kidney cancer. High values of sensitivity and specificity were obtained (**Figure 11**). Thus, ASL perfusion can be recommended for inclusion in the diagnostic algorithm for patients with metastatic brain lesions of unknown origin.

## Discussion

This study has demonstrated the role of ASL-perfusion in distinguishing brain metastasis from glioblastoma. In addition to this, we studied the blood flow in different subgroups of brain metastasis.

We observed statistically significant differences in intratumoral and peritumoral blood flow between glioblastoma and metastasis. Moreover, we calculated AUC, specificity, and sensitivity and found that for peritumoral blood flow, these values were 0.75, 68, and 73%, respectively, for the maximum TBF and 0.77, 58, and 91% for normalized TBF. For intratumoral blood flow AUC, specificity and sensitivity were 0.64, 70, and 60%, respectively, for maximal TBF and 0.66, 77, and 62% for normalized TBF.

Our results are explained by the biological characteristics of glioblastoma (4). Engelhorn et al. (17) and other studies (6) showed that the boundaries of glioblastoma do not coincide with the contrasted part of the mass on MRI. Tumor cells and alterations of the glial enviroment can be detected in the perifocal zone of increased MR signal in the T2 and T2-FLAIR. The researchers (17) have shown this through *in vivo* experiments in mice. All laboratory animals underwent an MRI study with contrast enhancement, then the brain was removed, and histological examination and immunohistochemistry were conducted, in which tumor cells and were found in the area of perifocal edema. Due to these features of glioblastoma growth, the term “perifocal edema” does not apply to the designation of the perifocal zone of an altered MR signal without signs of accumulation of a contrast agent; it is more correct to use the term “edema-infiltration” or “infiltrative edema” (18, 19).

We compared our results to other studies in the literature. Sunwoo et al. studied intratumoral and peritumoral TBF in 128 patients with GB and metastases (20).

For the peritumoral blood flow, we acquired results, which are in the same range—Sunwoo et al. obtained 64 and 89.7%, while in our work, values of 58 and 91% were obtained.

A comparison of intratumoral blood flow yielded sensitivity and specificity values of 92.1 and 43.6%, respectively. For intratumoral nTBF, our results were 77 and 62%.

The difference between the results can be due to the different composition of the group of metastases. In addition to that, methods of normalization and ROI selection are different—Sunwoo et al. performed normalization to gray matter values, while in our institute normalization is traditionally performed to white matter TBF. In the study by Sunwoo, at least two ROIs for each region were drawn and the average of the mean of each ROI was recorded (20) while in our study we manually put 4–6 ROIs in every slice with the tumor tissue and then chose the ROI with the highest TBF.

Lin et al. studied 52 patients with glioblastomas and metastases (21). These authors concentrated on ROI gradients in their work and on intratumoral and peritumoral blood flow.

For intratumoral blood flow, the authors did not obtain statistically significant differences. This factor is distinct from our work, probably due to the different composition of the metastasis group In the Lin's study the majority of the brain metastases were of the lung cancer origin.

For peritumoral blood flow adjacent to tumor, the authors obtained values of sensitivity and specificity of 64.29 and 83.33%, respectively. Normalized peritumoral blood flow numbers were 57.14 and 100%. In comparison, we calculated 68 and 73% for mTBF and 58 and 91% for nTBF. While these results are roughly in the same range (especially for the nTBF), the difference in the specificity for maxTBF could be due to the fact that ¼ of patients in the Lin work received steroids, which could affect the study results.

Also, these authors studied the gradient between ROI1 and ROI3 and calculated 92.86 and 100% sensitivity and specificity. Despite the fact that we were able to obtain statistically significant differences between ROI gradients in metastases and glioblastoma our ROC analysis yielded much lower results—59 and 71%.

In the work of Abdel et al. (22), the role of ASL perfusion in the differential diagnosis of glioblastomas and metastases in a group of 36 patients [n (GB) = 21, n (MTS) = 15] was studied. When comparing intratumoral blood flow, the authors obtained high sensitivity and specificity values of 86.7 and 95.2. A comparative analysis of peritumoral blood flow was carried out (ROI with a diameter of 0.5–2 cm was established within 1 cm of the contrasted part of the tumor) and sensitivity and specificity values of 86.7 and 90.5 were obtained. These indicators significantly exceed the values obtained by us and by other authors, which may be associated with a small sample of patients in this work and a different process of ROI selection.

To conclude, measuring peritumoral blood flow may be of greater diagnostic value due to better AUC, specificity, and sensitivity as well as better interstudy agreement.

Secondly, we studied blood flow in different subgroups of brain metastases and compared them with each other. A statistically significant difference was obtained between TBF in the lung cancer and melanoma subgroups and the lung cancer and kidney cancer subgroups. ROC analysis was also carried out.

The lowest mean blood flow was obtained in the metastatic colon cancer group. However, when comparing this group with others, no statistically significant differences in tumor blood flow were found, which may be due to the small number of patients in this group.

The highest blood flow values were obtained in the metastatic kidney cancer group, where all patients had clear cell carcinoma. When analyzing blood flow in these patients, two subgroups emerged: one with a very significant increase in blood flow compared to the white matter of the contralateral side (more than 10 times), and the other with low blood flow values, which practically did not differ from those in unchanged white matter, which is probably due to a pronounced necrotic component in the second subgroup.

In our study, the role of pCASL-perfusion in the differential diagnosis of various histological subtypes of metastases was investigated. We calculated the maximum TBF in lung, melanoma, kidney, and intestinal cancers and compared them to each other.

In the article by Dolgushin (23), the average values of blood flow using CT-perfusion in different histological types of metastases were calculated. The highest TBF in this work was obtained for melanoma metastases (113.99 ± 29.19). For the lung, breast, kidney, and intestine subgroups, values of 85.12; 92.05; 73.94; and 97.05 ml/100 g/min were calculated, respectively. Differences from this work may be due to different physical methods for measuring hemodynamic parameters (24). In the study by Qui et al. (25) where ASL perfusion was studied compared with CT with xenon, it was shown that the pCASL values are closest to the physiological values of blood flow, whereas with CT perfusion, these parameters may vary. The most significant differences with this work are presented in the values of blood flow in metastases of renal cell carcinoma. However, in our study, it was revealed that these metastases can be characterized by both very high blood flow and TBF values that practically do not differ from the white matter, which probably determines these differences.

Our study has several limitations. First, it was a retrospective study. Secondly, the study groups are not equal and, for example, patients with metastasis of colon cancer are not represented enough.

## Conclusions

In our study, we could obtain statistically significant differences between the intra and peritumoral TBF in glioblastoma and metastases. An interesting feature that could be used in making a diagnosis is the presence of zones with high blood flow in the peritumoral area of glioblastoma. This fact could be explained by the infiltrative nature of glioblastoma growth.

When studying blood flow in various histological subtypes of metastases, significant differences in TBF were found. High sensitivity and specificity values were obtained between the groups of melanoma and lung, lung and kidney, which suggests that pCASL-perfusion could contribute to identify the source of metastatic tumor.

## Data Availability Statement

The raw data supporting the conclusions of this article will be made available by the authors, without undue reservation.

## Ethics Statement

The studies involving human participants were reviewed and approved by the local committee of the N.N. Burdenko National Neurosurgery Institute. Written informed consent to participate in this study was provided by the participants’ legal guardian/next of kin. Written informed consent was obtained from the individual(s) for the publication of any potentially identifiable images or data included in this article.

## Author Contributions

Conceptualization, AB and KS. Methodology, AB. Software, EP. Validation, IP, NZ and AB. Formal analysis, AB, and SG. Investigation, KS. Resources, KS and SG. Data curation, AB and SG. Writing—original draft preparation, KS. Writing—review and editing, IP, NZ, and SG. Visualization, EP. Supervision, IP. Project administration, AB. All authors listed have made a substantial, direct, and intellectual contribution to the work and approved it for publication.

## Funding

This study was supported by the Ministry of Higher Education Agreement 075-15-2021-1343.

## Conflict of Interest

The authors declare that the research was conducted in the absence of any commercial or financial relationships that could be construed as a potential conflict of interest.

## Publisher’s Note

All claims expressed in this article are solely those of the authors and do not necessarily represent those of their affiliated organizations, or those of the publisher, the editors and the reviewers. Any product that may be evaluated in this article, or claim that may be made by its manufacturer, is not guaranteed or endorsed by the publisher.
